# MC64-ClustalWP2: A Highly-Parallel Hybrid Strategy to Align Multiple Sequences in Many-Core Architectures

**DOI:** 10.1371/journal.pone.0094044

**Published:** 2014-04-07

**Authors:** David Díaz, Francisco J. Esteban, Pilar Hernández, Juan Antonio Caballero, Antonio Guevara, Gabriel Dorado, Sergio Gálvez

**Affiliations:** 1 Dep. Lenguajes y Ciencias de la Computación, Universidad de Málaga, Málaga, Spain; 2 Servicio de Informática, Universidad de Córdoba, Córdoba, Spain; 3 Instituto de Agricultura Sostenible (IAS-CSIC), Córdoba, Spain; 4 Dep. Estadística, Universidad de Córdoba, Córdoba, Spain; 5 Dep. Bioquímica y Biología Molecular, Universidad de Córdoba (Campus de Excelencia Internacional Agroalimentario), Córdoba, Spain; University of California, San Francisco, United States of America

## Abstract

We have developed the MC64-ClustalWP2 as a new implementation of the Clustal W algorithm, integrating a novel parallelization strategy and significantly increasing the performance when aligning long sequences in architectures with many cores. It must be stressed that in such a process, the detailed analysis of both the software and hardware features and peculiarities is of paramount importance to reveal key points to exploit and optimize the full potential of parallelism in many-core CPU systems. The new parallelization approach has focused into the most time-consuming stages of this algorithm. In particular, the so-called progressive alignment has drastically improved the performance, due to a fine-grained approach where the forward and backward loops were unrolled and parallelized. Another key approach has been the implementation of the new algorithm in a hybrid-computing system, integrating both an Intel Xeon multi-core CPU and a Tilera Tile64 many-core card. A comparison with other Clustal W implementations reveals the high-performance of the new algorithm and strategy in many-core CPU architectures, in a scenario where the sequences to align are relatively long (more than 10 kb) and, hence, a many-core GPU hardware cannot be used. Thus, the MC64-ClustalWP2 runs multiple alignments more than 18x than the original Clustal W algorithm, and more than 7x than the best x86 parallel implementation to date, being publicly available through a web service. Besides, these developments have been deployed in cost-effective personal computers and should be useful for life-science researchers, including the identification of identities and differences for mutation/polymorphism analyses, biodiversity and evolutionary studies and for the development of molecular markers for paternity testing, germplasm management and protection, to assist breeding, illegal traffic control, fraud prevention and for the protection of the intellectual property (identification/traceability), including the protected designation of origin, among other applications.

## Introduction

The amount of genomic data is growing exponentially, due to the advances in technology and the evolution in the so-called “Next-Generation” Sequencing (NGS), including the latest second- and third-generation equipment. Thus, the former bioinformatics tools from the genic era are evolving to handle the current genomic data. These include alignment algorithms for sequence comparison like the Needleman-Wunsch [1] pairwise global-alignment algorithm, which has evolved both from a biological point of view, as the Smith-Waterman local aligners [2] or Gotoh affine gaps [3], and also from a computational point of view, as the Hirschberg linear space approach [4] or Driga FastLSA [5].

Yet, these computational improvements in pairwise-alignment algorithms cannot properly handle long or very long sequences, like some partial or complete chromosomes and genomes. Thus, some heuristic strategies have been developed to overcome the large amount of memory and execution time required to align such sequences. The Fast Alignment Sequence Tools (FAST)-All (FASTA) [6] and Basic Local Alignment Search Tool (BLAST) [7] are examples of this trend. The heuristics are able to obtain approximate alignments with acceptable accurate results in much less time, so they can be useful to compare long sequences. Nonetheless, the quality of the alignment may be compromised in some instances [8], and therefore non-heuristic aligners may be needed to guarantee the optimal alignment from a computational point of view.

Additionally, the “optimal” pairwise aligners can be ported to new computing architectures to overcome the previous limitations. Though an alternative to speedup algorithms is to use supercomputers, grid computing and clustering using several nodes [9], more affordable options are available nowadays. That is the case of the multi-core architectures, where several threads or processes run independent code in parallel, albeit requiring the programmers to develop new approaches when porting, adapting and optimizing the existing alignment algorithms to the new parallel architectures. In fact, the number of cores is increasing exponentially, giving birth to the concept of “many-core” architectures, where tens, thousands and even millions of them are available.

**Figure 1:**
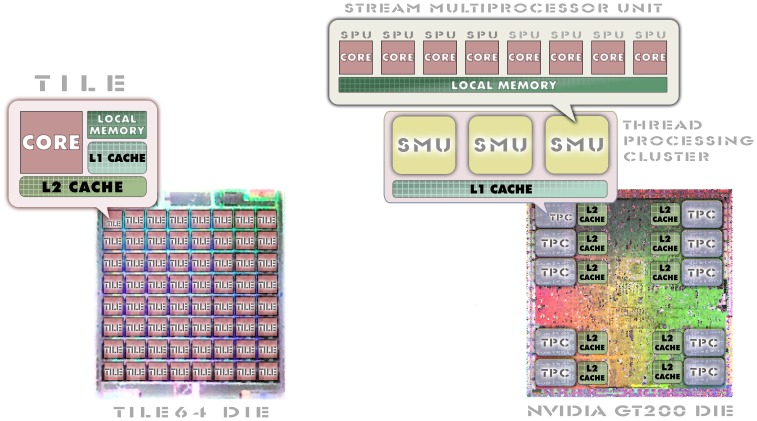
Block diagram of many-core CPU and GPU architectures. The shown dies are boarded on nVidia GT 200 GPU series and Tilera Tile64 Peripheral Component Interconnect express (PCIe) cards. The cores in the many-core CPU architectures are interconnected through a high-bandwidth mesh, whereas the ones in the many-core GPU are isolated.

At this point it is important to distinguish between multi-core and many-core microprocessors. Thus, in many-core architectures, the execution cores are small Central Processing Units (CPU) with fewer resources than a standard multi-core CPU; so in order to extract the full potential of the former, specific parallelism strategies for them should be developed. Besides, the many-core microprocessors can be classified into two different groups: many-core Graphical Processing Units (GPU), and many-core CPU. The former ones have thousands of Stream Processors Units (SPU), sometimes named cores, distributed in a hierarchical way: a GPU has several Thread Processing Clusters (TPC), where each one consists of an array of Stream Multiprocessor Units (SMU), having each one eight SPU. The resources are shared between the SMU in these models. On the contrary, the many-core CPU microprocessors have a matrix of uniform CPU with their own resources, usually interconnected via a high-throughput network. A graphical comparison of both architectures can be seen in Figure 1. Unfortunately, such architectural differences require also different programming methodologies, being the Compute-Unified Device Architecture (CUDA) a specific model to exploit the parallelism in General-Purpose GPU (GPGPU) [10] and the standard C being usually deployed in many-core CPU architectures. In the field of cluster computing, the C language is sometimes complemented by some kind of standard or proprietary Application Program Interface (API), to extend some parallelism abilities like message passing, shared memory or abstract channel communications.

There are experimental and commercial many-core CPU models. They include the Adapteva Epiphany IV with 64 cores [11], the Sun Microsystems UltraSPARC T2 Pro with eight cores and eight threads per core [12], and the models from Intel, the leader company in this research field with the Many-Integrated Cores (MIC) architecture. At first, they created the Intel Terascale microprocessor with 80 cores [13], and then experimental models like the Single-chip Cloud Computing (SCC) [14] with 48 cores and the Knights Ferry [15] with 32 cores. From the experience obtained from this models and the Larrabee hybrid CPU-GPU project, a new model codenamed Knights Corner and eventually named Xeon Phi was developed with 61 cores and 22 nm 3-D tri-gate process fabrication process. The second generation of Xeon Phi products (Knights Landing) is expected in the near future as a coprocessor or a host processor (CPU), manufactured with 14 nm node technology and second-generation 3-D tri-gate transistors.

Following this trend, Tilera has developed a many-core CPU architecture known as the Tile64 microprocessor, being a 90 nm Reduced Instruction Set Computing (RISC) System-on-Chip (SoC) microprocessor with 64 general-purpose CPU called tiles, each one being able of running an independent operative system (Linux). Each core runs at 500–866 MHz and can reach a global 0.166 Tera-Instructions Per Second (TIPS). The tiles are interconnected through a high-throughput network called intelligent Mesh (iMesh) with 31 Terabits per second (Tbps) bandwidth. As seen in Figure 1, each tile contains its own Level 1 (8 KB for data and 8 KB for instructions) and Level 2 (64 KB) caches. Furthermore, all the Level 2 caches become a larger Level 3 common cache [16]. Lately, Tilera has announced a future branch of microprocessors called Tile-Gx [17] with 16 to 100 cores using a 40 nm fabrication process.

Thus, thanks to the emerging many-core technologies, new approaches are available to address the increasing demand of computational power. This allows to tackle the bioinformatics analyses required for the exponentially increasing data generated by the new sequencing methodologies. That is the case of the previously mentioned Needleman-Wunsch (NW) [1] and Smith-Waterman (SW) [2] pairwise aligners, where GPU parallel approaches have been presented by Manavski [18] and Liu [19]. However, most of these implementations are designed to align a single query sequence against a huge number of small sequences like those from peptides (eg., proteins), yet cannot align sequences longer than 59,000 residues. This is due to the intrinsic “Single Instruction, Multiple Data” (SIMD) characteristics of the GPGPU, where pipelining allows a great speedup factor, yet intense memory usage may lead to bottlenecks.

Indeed, although some many-core CPU microprocessors with tens of cores have recently arisen, there are only a few bioinformatics developments deployed in such architectures, as the ones that we have previously published for the Tile64 microprocessor [20]–[22]. Such developments were carried out with TILExpress-20G cards including the Tile64 microprocessor and 8 GB of RAM, for which we have empirically demonstrated some limits: 7.8 GB for Solid State Disk (SSD), 2.8 GB for local memory and 1.9 GB for shared memory. Such cards have high-bandwidth communication ports and a very good performance per watt [20]. Among others, we have developed a parallel version of the NW/SW algorithm, named Multicore64-NeedlemanWunsch/SmithWaterman (MC64-NW/SW), using a FastLSA strategy [5], which we have thoroughly optimized taking into account the hardware and algorithm features and peculiarities. This implementation achieved a gain of ∼1,000% against the same algorithm on a x86 multi-core architecture, allowing to align sequences of one Megabase (Mb) length in 23 minutes approximately [20], [22].

In this report, we have extended our work to the popular Multiple-Sequence Alignment (MSA) algorithm known as Clustal W. The new parallel version of Clustal W exploits the high-throughput parallelism in many-core CPU architectures, optimizing the most time-consuming stage of the original algorithm. This has been accomplished taking into account both the existing parallel versions of the Clustal W for multi-core and many-core GPGPU architectures, as well as our previous experience when developing the parallel MC64-NW/SW [20]. Thus, the new algorithm, called MC64-ClustalWP2 for Many-Core64-Clustal W Phase 2 (of parallelization), was implemented for the Tilera Tile64 microprocessor, as described below.

Different benchmarks were run to test the new algorithm against the previous developments and to quantify its performance. Since the MC64-ClustalWP2 algorithm was developed to align relatively large sequences, for instance, from 10 kilobases (kb) to 300 kb, it was tested with a family of organisms whose genome lengths fall into such a range. It should be noted that this approach would require an excessive execution time in a multi-core system or would not even be allowed in a GPGPU architecture, due to the length of the sequences. The MC64-ClustalWP2 source code is available under the General Public License (GPL) license at the <http://galactus.uma.es/manycore> web site [23], where the algorithm can be also remotely invoked through web services.

### Multiple-Sequence Alignments

The multiple-sequence alignments allows the comparison of two or more sequences, in contrast to the pairwise aligners like Smith-Waterman, which are limited to just a couple of them. Therefore, the former are particularly useful to identify identities (similarities) and divergences (differences) between many sources, allowing to build evolutionary phylogenetic trees (dendrograms). Thus, the identification of small and large variations, mutations or polymorphisms like base changes and insertions/deletions (indels), duplications or amplifications, recombinations and rearrangements like translocations can be exploited to develop molecular markers for identification, including germplasm management and protection, paternity testing, marker-assisted selection and breeding, illegal traffic control, fraud prevention and traceability. This can be applied to the Protected Designation of Origin (PDO), which is a label of food products from some geographical areas, showing particular organoleptic or otherwise desirable characteristics, conferring them a higher quality than similar products from other sites.

However, generating a Dynamic-Program Matrix (DPM) in the sense of “optimal” pairwise aligners, but for more than two sequences, means a Nondeterministic Polynomial (NP) complete complexity problem in computation, and therefore no fast solution to it is known by definition. For this reason, several heuristic strategies were developed to simulate the behavior of the n-dimensional matrix, using a batch of pairwise alignments and simpler DPM. That is the case of MULTAN [24], being a Waterman [25] or Clustal [26] method. The latter has become very popular, due to its original performance and further developments and improvements. The Clustal algorithm is based on a progressive-alignment strategy of all the sequences, aligned by the order determined by a previously-calculated phylogenetic tree, generated from the similarities and differences among the sequences in an all-vs-all comparison matrix.

The first version of MULTAN was programmed in the FORTRAN language, but later on it was re-programmed in the C language, and a few new functionalities were added, like the generation of a dendrogram in the final stage [27]. More notable changes were introduced in the new Clustal W [28], which received the “W” for “weighting”, since in this version the sequences were weighted in order to increase the sensibility of the algorithm. Later on, the version 2 was ported into C++, including two new minor-relevant features [29]. Besides the Clustal, there are other multiple-sequence alignment algorithms, like the T-Coffee [30] and MUSCLE [31], which may obtain accurate-enough results for large sequences using heuristics, modifying the progressive alignment method and adding new refinements.

Furthermore, as with pairwise aligners, quicker MSA heuristic methods which do not rely on a DPM have been also developed, as the Multi-LAGAN general anchoring-based method [32]. In addition, new multiple-sequence alignment approaches have been published, including the so-called genome MSA methods, capable of dealing with very long similar sequences, like MGA [33] or MAVID [34]. These methods are very fast, but they are usually not accurate when the aligned sequences have highly polymorphic regions (e.g., with high mutation-rates). Therefore, they can be useful to align restricted or local similar regions, but not to globally align any set of sequences [35]. More recent methods like the progressive Mauve [36] are able to deal with some sequence variations (like rearrangements), but not with all of them, as is the case for duplications [37]. A further review of the most important MSA algorithms can be found at [38].

On the other hand, the Clustal W design is divided into three main stages: the distance matrix generation, the guide tree generation and the progressive alignment. It can be easily determined that the most time-consuming stages are the first and the last ones [39]. Indeed, they require most of the computation time as the number of sequences or their length increase. Therefore, their optimization represents the first theoretical choice to improve the algorithm performance. Thus, the Clustal W has been parallelized for SGI computers [40], multi-core platforms using threads [41], the hybrid multi-core Cell chip [42] and scalable clusters using the Message Passing Interface (MPI) [43]. However, none of them has really exploited the parallelism in the third stage, as it is pointed out in the next section.

### Clustal W Behavior Analyses

In order to properly optimize the Clustal W parallelization, each stage of the algorithm should be carefully analyzed. Traditionally, the Clustal W has been used to align short sequences of nucleic acids (DNA or RNA) and peptides, with high-performance implementations of Clustal W exploiting parallelism to obtain the best results for larger number of such sequences. However, as the MC64-ClustalWP2 focuses on longer DNA sequences (for instance, from 10 kb to 300 Mb), the parallelization and optimization strategies should be different.

**Figure 2:**
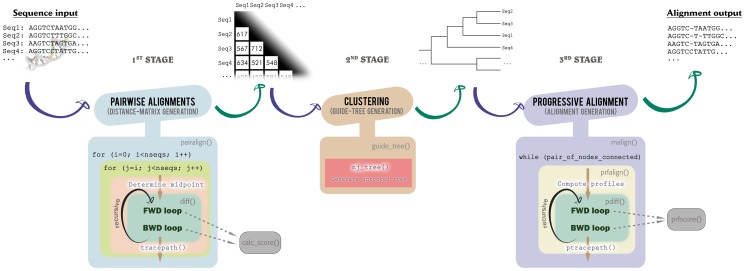
Global behavior of the Clustal W algorithm and its three main stages. The top part shows the three main stages of the Clustal W showing both the data used and generated in each one. In the bottom part, the functions and high-level pseudo-code reveal the similarities between the most time-consuming and representative parts of the algorithm.

As previously noted, the Clustal W is divided into three main stages, shown at the top of Figure 2. The first stage fulfills the cells of a distance matrix with a score that represents the distance between every pair of sequences. Each score is calculated running a pairwise-alignment operation. The second stage generates a guide tree using a clustering method, like the Neighbor-Joining (NJ) [44] and, finally, the third stage progressively generates the multiple alignment, following the topology of the guide tree.

The pairwise alignments of the first main stage are calculated using the local Myers-Miller algorithm [45], a Smith-Waterman pairwise aligner that implements affine-gap penalties [3]. Its space complexity is linear, at the expense of doubling the execution time when compared to other standard pairwise aligners. The multiple alignment of n sequences requires to complete the distance matrix, performing n/2*(n−1) alignment operations between each pair of sequences. There are no dependencies between them, so they can be executed in parallel. The MPI-alike implementations for Personal Computer (PC) clusters usually assume this coarse-grained approach [46]. A more fine-grained approach is used by the MSA-CUDA [47], as each pairwise alignment is partially distributed among several executing threads, thus obtaining two levels of parallelism. Each alignment returns a score value, which is then stored into the corresponding cell of the distance matrix. In addition, the actual pairwise alignments are also required by Clustal W to fine-tune the distance matrix values.

The clustering stage builds a phylogenetic tree from the previously calculated distance matrix, returning an unrooted tree with the evolutionary distances between branches. This is the less time-consuming stage [39] and, when working with only a few sequences (n<100), it can be considered negligible, as its complexity only relies on the number of sequences to align. In any case, the algorithm used for this node-clustering phase is the Neighbor-Joining (NJ) method [44], which has been previously parallelized for GPU many-core systems [48]. Other methods like the Unweighted Pair Group Method with Arithmetic Mean (UPGMA) [49] can also be used.

Finally, the progressive-alignment stage globally aligns sequences in an iterative way, following the path described by the unrooted-guide-tree topology. This stage starts aligning the closest pairs of leaf sequences. Then, the resulting pairwise alignments must be aligned again, following the closest path given by the guide tree. This high-level alignment strategy is called profile-profile alignment, where a profile is obtained from a previous intermediate of the multiple alignment, following a path from the leaves to the top of the tree. Thus, a profile alignment presents a more advanced scoring system and is more computationally expensive than a common-pairwise alignment. Furthermore, dependencies in the tree do not allow parallelizing all alignments, as occurs in the first stage. Therefore, only independent parallel-branches of the tree can be simultaneously executed, and thus may be restricted by the tree shape. This low-parallelization factor is determined by the log *n* in a well-balanced tree of *n* nodes for the best-case scenario. In a deeper level, the core of the aligner can be divided as well into two independent loops to be run separately, corresponding to the forward and backward loops [50]. Some implementations take advantage of this fact to run both loops in parallel, as the ClustalW-MPI [43]. The result produced by this stage is the full alignment.

### A New Strategy to Parallelize Clustal W: MC64-ClustalWP2

In order to exploit the parallelism in the many-core architectures, the tasks to be executed must be spread among all the available cores, which should communicate between themselves at a high speed. Thus, the first step carried out in this work was considering Clustal as three main tasks to be independently parallelized. In addition, as we focus on aligning a few long sequences, the second stage can be ignored, because its execution time can be considered negligible. An exhaustive analysis of the Clustal W allowed us to identify the progressive alignment source-code that should be rewritten, in order to have a structure very close to that of the pairwise-alignments stage. A bird's eye Clustal W structure of such approach is shown at the bottom of Figure 2.

This chart reveals the inherent similarity between the first and the last stage, where the alignment of a pair of sequences is replaced by the alignment of a pair of profiles. These profile alignments make use of a more-advanced scoring system, and they are far-more computational expensive. In particular, the score calculus function, prfscore(), the hot spot of the aligner, is a time-consuming vector multiplication (whereas in the first stage, the corresponding operation is a straightforward access to an array cell).

### A new parallel approach of the pairwise-alignments stage

In order to parallelize the first ClustalW stage in a previous work, we replaced it with an iterative call to the MC64-NW/SW pairwise aligner [21], obtaining more than a 60% performance speedup against the ClustalW-MPI executed in a quad-core Xeon system [43]. The resulting algorithm, named MC64-ClustalW, was a coarse-grained strategy, as only individual alignments were parallelized. Besides, a considerable percentage of the tiles were idle at the beginning and at the end of each alignment, due to the wave-front growth behavior of the algorithm. Furthermore, only a tile was used in the backward stage of a pairwise alignment, being therefore a waste of resources.

Following the Tile64 terminology, a geometry is a set of adjacent cores that forms a rectangular shape, behaving as an independent subset of the Tile64 chip from a functional point of view. The Tile64 can be partitioned into several geometries, but any tile can only belong to a particular geometry at a given time. In the present work, we propose a much more advanced and fine-grained approach, being a new phase in our efforts to improve the performance of Clustal W, in which the parallelism is exploited at two levels: i) several alignments are calculated simultaneously in different geometries of cores; and ii) inside each of them, the alignment is computed in parallel by all the cores contained in it.

To achieve this new run-distribution requires a higher-level controller to schedule alignments among all groups of geometries. This new design unrolls the two main loops in the pairalign() function, and replaces the original linear-space approach with a call to the MC64-NW/SW which, in turn, uses an advanced FastLSA strategy. With this unrolling method, each pairwise alignment is assigned to an available geometry, in the same way as our previous MC64-NW/SW works [20]. Therefore, the number of pairwise alignments that can be simultaneously calculated corresponds to the number of geometries into which the Tile64 core-array is divided. Nonetheless, forward and backward stages of the pairwise alignment are separated: geometries of *n* workers calculate the full DPM for the forward stage (*F-xx geometries* from this point on), but geometries of only one worker perform the backtracking and alignment generation (*B-xx geometries* from this point on). Thus, several working geometries of these two classes collaborate to calculate all the alignments, taking the most out of every available tile. Meanwhile, the high-level controller schedules and manages all the pending jobs. Furthermore, the temporary grid-cache data must be shared between their geometries, in order to communicate a forward stage job with its backward stage counterpart.

### A new parallel approach of the progressive-alignment stage

With MC64-ClustalWP2 we propose as well a new parallelization strategy for the progressive-alignment stage. As stated above, Figure 2 shows that the progressive alignment is actually a Myers-Miller-like alignment algorithm, whose two main loops can be unrolled in a similar way as the pairwise alignment was done. Therefore, we propose to transform the linear-space recursive Myers-Miller into a quadratic-space sequential Needleman-Wunsch. In both cases, the corresponding progressive affine-gap algorithm must be applied. In a similar way as with the first stage, this sequential dynamic-programming strategy has been parallelized using the FastLSA approach, in order to reduce the constant multiplicative factor.

However, two levels of parallelism cannot be achieved in this progressive-alignment stage, because each alignment profile depends on the previous ones; these dependencies are given by the guide tree. Our parallel approach transforms the prfalign() function into a new parallel program, using the same FastLSA strategy to distribute a single alignment to all the available tiles in the many-core system. Thus, applying the wave-front parallel strategy and selecting an optimal *k value*, the speedup will depend on the number of available processing resources, as long as the scalability factor is very close to that of our previous MC64-NW/SW [20].

In this design, a controller tile is in charge of the management of the grid cache and the distribution of jobs among the rest of available worker tiles. The profiles of the original Clustal W are still used, so the profile initialization is performed by the controller tile, whereas the workers call the original prfscore() calculus-function profile. The new implementations of both the pairwise and progressive aligners, as well as their relative speedups, are discussed in the next sections.

### Deployment of the MC64-ClustalWP2 on the Tile64 Many-Core Microprocessor

The main asset of the many-core CPU Tile64 System-on-Chip is the execution of parallel programs, due to its 64 cores/tiles running at 866 MHz in a TILExpress-20G card. The drawback is that inherently sequential code is poorly performed by a single tile. Considering that the Clustal W contains purely sequential blocks of code, yet being potentially parallelizable, we have based our implementation on a heterogeneous programming model. In such an approach, the code is commonly executed in the host CPU, using many-core GPU cards as code accelerators, effectively exploiting the best complementary potential of each kind of microprocessor. Different standards and programming methodologies have been proposed to unify and schedule the tasks in these systems, like the OpenCL [51]. Albeit, no one supports the Tile64 architecture yet, due to the novelty of the many-core CPU technologies.

The Clustal W 1.83 source code, written in the C programming language, has been carefully adapted to support the heterogeneous programming approach, generating a x86-Tile64 implementation of the MC64-ClustalWP2 parallel algorithm. Thus, the MC64-ClustalWP2 is composed by three executables: one runs in the host and two run in the Tile64. The main one is executed in the host; it processes the input parameters, sets the internal variables and orchestrates the three stages of the algorithm, eventually producing the multiple alignment. The three stages of the Clustal W are scheduled between the host CPU and the many-core CPU, as follows:

- The pairwise alignments are launched in the Tile64 environment, following the schema previously described. The pairwise-alignments overseer is the previously named “high-level controller”, a Perl script launched in the host to supervise the work distribution among the geometries.
- Once every pairwise alignment and its score are obtained, the host performs the clustering algorithm, whose execution time is negligible compared to the other stages, since the number of sequences is reduced. Therefore, the parallelization is not worth the effort in this stage. The result is the phylogenetic tree.
- Again, each profile alignment of the progressive-alignment stage is executed in the Tile64 microprocessor, following the job distribution previously stated. Every intermediary result is processed by the host, in order to calculate the next one, until the final multiple alignment is obtained.

**Figure 3:**
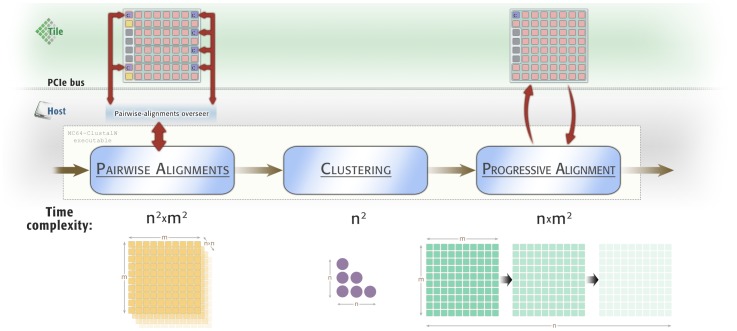
Heterogeneous programming model of the MC64-ClustalWP2 and each stage complexity. The executable algorithm is run in the host machine: the pairwise-alignments stage communicates with an external host program, which schedules the alignments among tiles. The clustering stage is executed in the host, and the progressive-alignment stage is controlled by the host, by calculating each profile alignment in the Tile64 microprocessor. The time complexity of each stage is shown in the lower part.

Therefore, this hybrid work distribution exploits both architecture strengths, thus running the sequential code in the PC host and the parallel code in the Tile64 microprocessor. The complete workflow can be seen in the Figure 3. Following this approach, the implementation requires communicating both platforms for pairwise and progressive alignments.

### Implementation of the new pairwise-alignments stage

The MC64-ClustalWP2 pairwise aligner design requires two different job classes to be run in the many-core platform; they deal with the forward and backward stages, respectively. In order to balance the developing effort versus the performance gain, the starting point was our local MC64-NW/SW pairwise aligner [20], where the necessary modifications were carried out to adapt it to the new requirements.

Both stages were decoupled, but keeping the same controller-worker strategy. So, in order to run any job, at least two tiles are needed in each stage: one acting as the stage controller and the other executing the code of the worker stage. With this approach, the same executable runs a forward or a backward stage, depending on a flag parameter: the forward stage is launched on a *F-xx geometry*, whereas the backward stage is launched on a *B-xx* one. This requires to share data between the two controllers of the same alignment (now decoupled), by temporarily storing the grid cache and the internal variables in the TILExpress-20G card 8 GB SSD file system. On the other hand, the original MC64-NW/SW used 6 bytes per cell, since gap penalties are never greater than 128 [5]. However, the pairwise alignment in Clustal W uses bigger values, so the grid-cache cell structure was modified as well to support it. Now, 12 bytes are used and the absolute values are stored instead of the relative differences in the case of the two auxiliary matrices. This is translated into more memory/file-system usage and less performance, due to the Tile64 instruction characteristics. As a collateral effect, the optimal *k values* calculated in the MC64-NW/SW parallelization are invalid and must be recalculated.

Taking into consideration that four cores are reserved for the internal management of the chip and communications with the host, the 60 remaining available cores are distributed into a number of static geometries. Different empirical tests have demonstrated that the optimal geometries to use are four forward stage geometries of 7×2 tiles and two backward stage geometries of 2×1 tiles. The rationale of such result is that the forward stage is far more computationally expensive than the backward one. Nonetheless, when the number of pairwise alignments is between five and eight, it is better to use eight 7×1 F-xx geometries, because this optimizes the workload by avoiding a second batch of alignments with idle geometries.

To achieve two levels of parallelism, a “high-level” controller is needed to manage several alignments simultaneously; this controller appears in Figure 3 with the name Pairwise-alignments overseer. This controller runs in the host because it must communicate with the tile-monitor command-line tool which Tilera provides in its Multicore Development Environment (MDE), in order to manage the tile geometries. To take apart this functionality from the Clustal W source code, this task is carried out by a Perl script, which is called from the host main executable with the proper parameters: sequences to align, scoring matrix, etc.

Although other kinds of communications would be possible, like a telnet access, a client-server approach using the available Ethernet ports or a serial console, the communication between the Perl script and the Tile64 is performed via the more straightforward tile-monitor command line tool: when the Perl script starts a new tile-monitor session, it takes input/output communication channels through which the commands and results are sent. The Perl script manages pending jobs and available geometries, in order to schedule workload. Finally, when every pairwise alignment has been calculated, the MC64-ClustalWP2 pairalign() function gets all of them from the Perl script. Starting from them, the scoring matrix is computed as the original Clustal W does.

### Implementation of the new progressive-alignment stage

The implementation of the progressive aligner requires as well communicating the MC64-ClustalWP2 program, run in the host, with the parallel code executed in the Tile64. However, an intermediate script is not needed this time, as long as the algorithm has only one level of parallelism (every tile works for the same profile alignment and, thus, no fine-grained tile-monitor control is required).

This stage is controlled by the host main executable, which launches each profile alignment on the Tile64 environment. To do this, many internal variables must be shared between these both hardware environments. In total, 37 different variables and structures are needed, in order to rebuild the profiles and run the algorithm in the Tile64; other five are updated and returned to the host. To perform these communications and, at the same time, to avoid spreading dependencies throughout the code, we have built an intermediate layer library, called MC64-NWProfile_ParameterManagement, that encapsulates data structures and communication functions. This layer carries out communications using properly-formatted plain-text files. This facilitates uploading the required data into the Tile64, as well as download from it when required. In addition, the MC64_ParameterManagement provides the implementation of the profile-alignment algorithm, which is executed in the Tile64 microprocessor, as seen in Figure 3. Thus, the Clustal W malign() remains nearly the same, but the prfalign() function has been replaced by the needed calls to the parameter management library functions, which wait for the Tile64 program to finish before continuing.

The profile alignment algorithm for the Tile64 microprocessor has been developed starting from the MC64-NW/SW code, and applying several major changes to align profiles instead of sequences. For internal purposes, we have called MC64-NWProfile this new sub-algorithm, due to its global behavior. Each profile is a bi-dimensional structure of 35×m, where the height (35) is determined by all the possible residues plus gaps, whereas the width (m) is determined by the profile length; i.e., the length of the globally aligned sequences in the block. The progressive alignment is based on the alignment of these structures, with the prfscore() function determining the score for position i,j of the DPM from the vector multiplication of the profile1[i] row and the profile2[j] row.

Firstly, the MC64-NWProfile computes the profiles using the internal variables passed to it. Once calculated, the algorithm follows the same controller-workers approach than the MC64-NW/SW, using a similar wave-front parallelism. However, the prfscore() is now a vector-multiplication operation instead of an indirect-access operation. Additionally, the cells are 12 bytes instead of 6 bytes, so the job-partition optimal *k values* for each pair of profiles are completely different from those calculated in the pairwise-alignments stage. Thus, new optimal *k values* for significant profiles lengths must be pre-calculated, and, when a pair of profiles is to be aligned, the actual *k value* is interpolated from these ones.

To calculate a cell's content in this parallel approach, each worker requires accessing to the profile structures to compute the scores and alignment. However, for relatively long sequences, each profile can take several megabytes (for example, to align 200 kb sequences, each profile requires 24 MB), so a new problem arises: the location of these two profiles in memory. A direct approach to overcome such limitation is to store a single copy of the profiles in the shared memory, being accessible by both the controller and every worker. Yet, unfortunately, the shared memory access is very slow (can consume tens of CPU cycles) in comparison to the local memory access. Therefore, a better approach is to copy the profiles into the local memory of each tile. This simple modification represents an 8x speedup in execution time, though it requires much more memory (following the 200 kb example, 24 MB×2×59 workers  = 2.8 GB), and thus restricts the range of valid *k values*. Indeed, both the grid cache and the profile copies coexist in the available 8 GB (used as SSD shared and local memory). As a consequence, the available memory to store the grid cache decreases using this approach, and then, a low *k value* may produce a memory overflow.

In contrast, the MC64-NWProfile obtains a better performance with lower *k values*. Therefore, an intermediate approach is to only copy the necessary fragments for the current job to the worker local memory. Hence, when a worker receives a new job, it brings these fragments of the profiles to the local memory and when finished, it frees the memory. This approach opens the door to use small *k value* sizes, though the handicap is that the same fragments are eventually copied in the same worker several times for each alignment. In spite of this, using low *k values* instead of bigger ones, allows obtaining an additional 1.5x overall speedup, as it has been empirically demonstrated (data not shown). Finally, when the alignment is obtained, the local Clustal W variables are modified and returned to the host, which generates the intermediary multiple alignment and continues the process, following the guide-tree topology to progressively generate the next alignments.

## Results and Discussion

Once the algorithm was developed and implemented using the approach stated above, the MC64-ClustalWP2 was tested to benchmark its performance against the original algorithm and other parallel implementations, using different architectures and approaches. At first, we launched stress tests with ten sequences of different sizes and measured the speedup of the MC64-ClustalWP2 against other Clustal W implementations. A further test was carried out to analyze families of long sequences, which are aligned in much less time than with other general global MSA strategies, as discussed below.

### Heterogeneous Tile64/PC MC64-ClustalWP2 speedup tests

In order to test the relative performance of the new MC64-ClustalWP2 parallel strategy implementation with the many-core Tile64 microprocessor, the original Clustal W algorithm and other parallel implementations were compared using a set of sequences with different sizes. In particular, the tested algorithms were the Clustal W 1.83 (originally written in C language) [28] and two parallel implementations: the ClustalW-MTV (the latest version of MT-ClustalW for multi-threaded systems) [41] and the ClustalW-MPI (focused on multi-core systems and PC clusters using the MPI library) [43]. The Clustal W many-core GPU implementations, as the MSA-CUDA [47], were excluded from this test, since they are unable to align such large sequences. The dataset used for the experiment is composed of 10 sets of artificial sequences whose lengths range between 25 and 300 kb. The alignment of hundreds or thousands of sequences with lower lengths is not the focus of our parallel approach (and neither its strength), so it is not worthy to carry out any test on such sets of sequences. In these cases, the scientist may choose from a different set of tools specifically developed for the purpose, including the above-mentioned GPU implementations and programs like MAFFT (the PartTree algorithm [52]) and Clustal-Omega [53].

**Figure 4:**
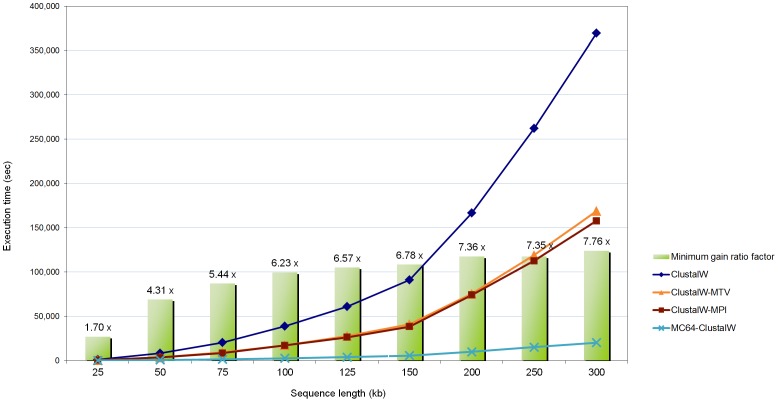
Execution time of the alignment of sets of ten randomly-generated sequences of different sizes, using Clustal W implementations. The tested algorithms are the original Clustal W 1.83, the MC64-ClustalWP2 for Tile64 and PC, and two parallel implementations for x86 systems (MT-ClustalW and ClustalW-MPI). In addition, the minimum gain ratio of the MC64-ClustalWP2 against all the other implementations is exposed for each set of sequences.

All of the algorithms tested were run in the same environment (Intel Xeon Quad Core 2.0 GHz PC with 8 GB of quad-channel DDR2 memory), providing the same results but with rather different execution times. The MC64-ClustalWP2 used the Tile64 microprocessor on-boarded in a TilExpress-20G card with 8 GB RAM. The execution time and minimum-gain ratio of the MC64-ClustalWP2 algorithm against the other ones is shown for each set of sequences in Figure 4.

**Figure 5:**
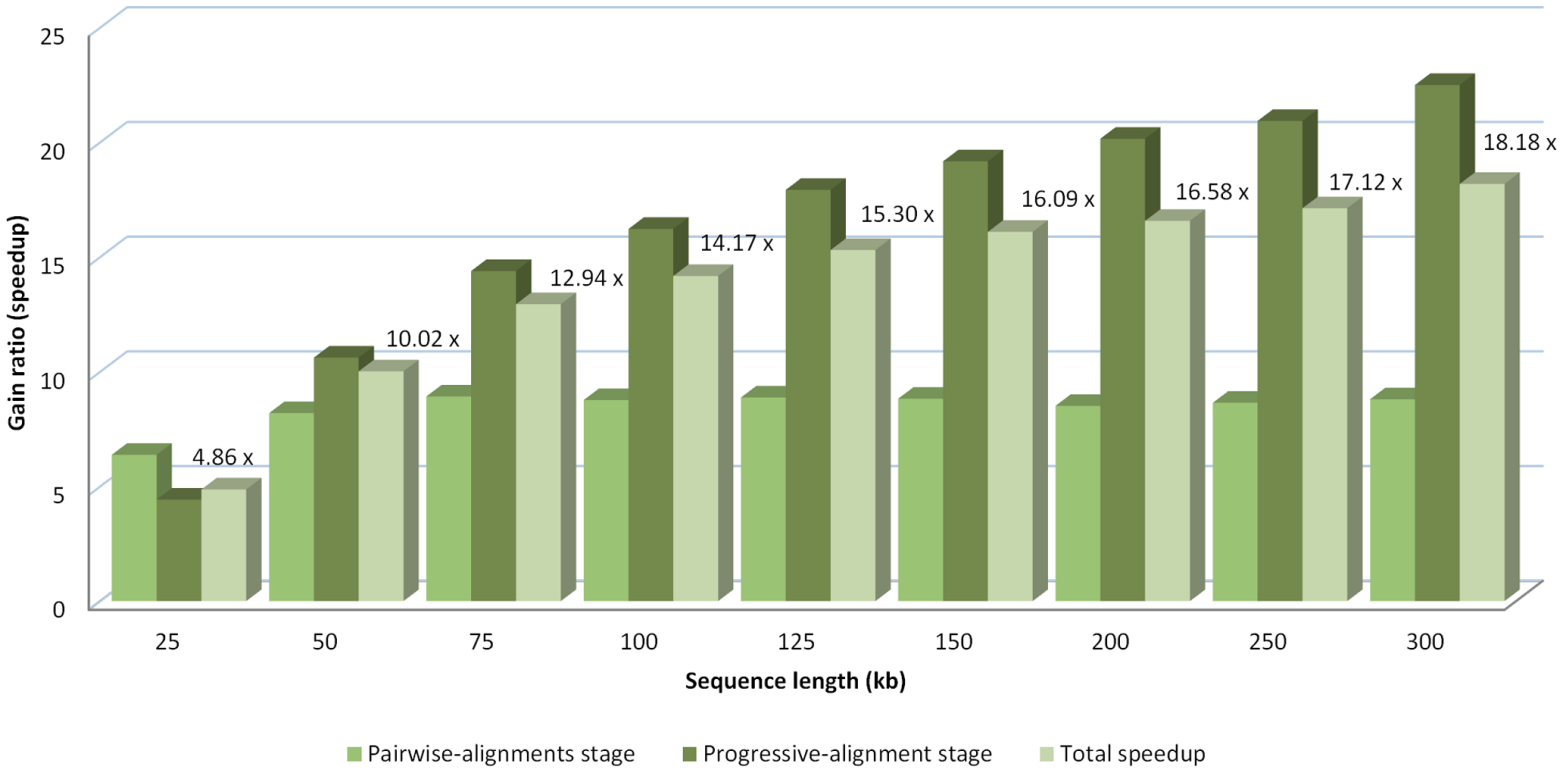
Speedup of the MC64-ClustalWP2 against Clustal W 1.83. The speedup is shown both for each relevant stage and for the full execution when aligning sets of ten randomly-generated sequences of different sizes.

**Figure 6:**
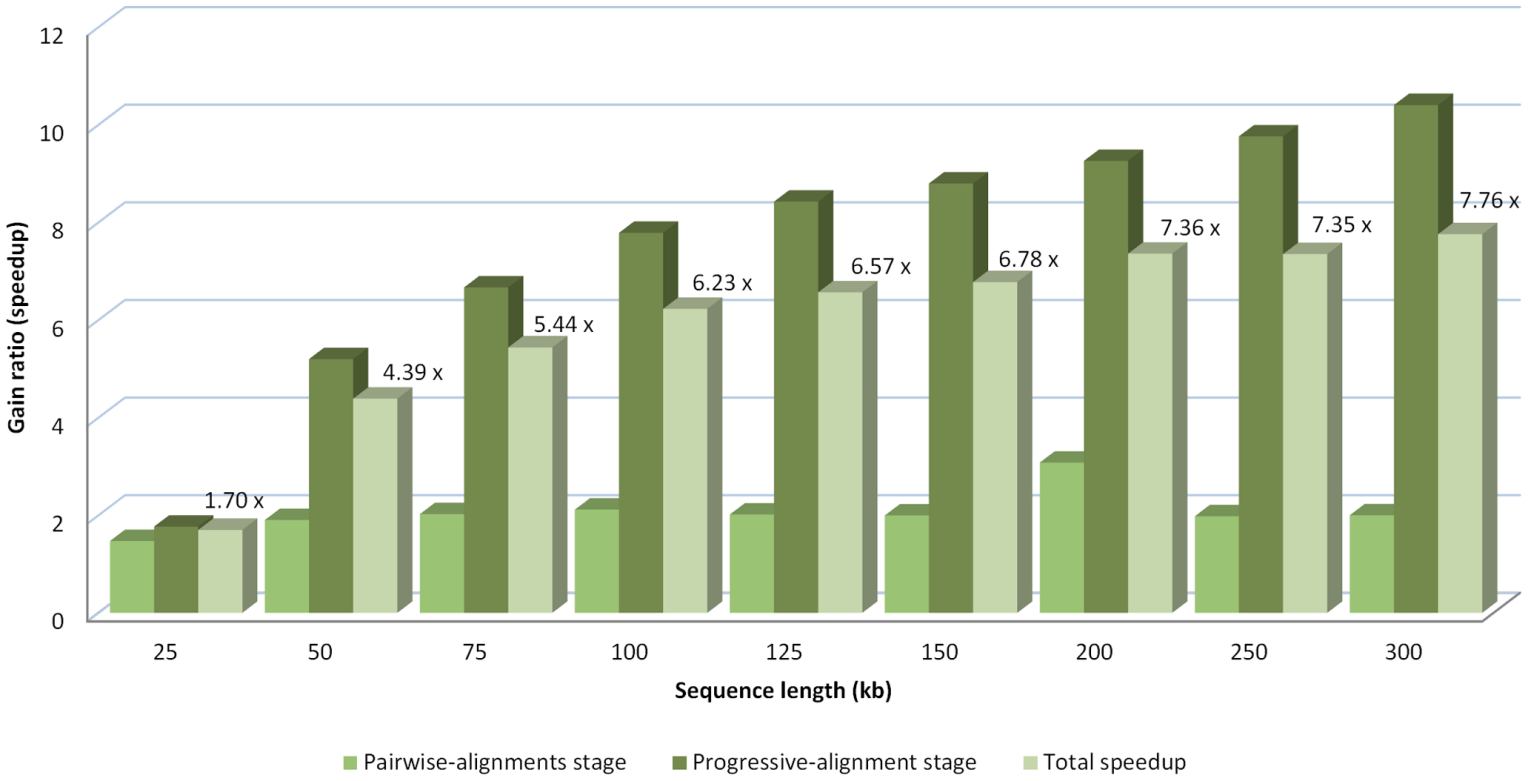
Speedup of the MC64-ClustalWP2 against ClustalW-MPI. The speedup is shown both for each relevant stage and for the full execution when aligning sets of ten randomly-generated sequences of different sizes.

The results reveal the best performance of the MC64-ClustalWP2 against every other implementation. This tendency increases with the length of the sequences, because a higher parallelization factor is obtained. Thus, the MC64-ClustalWP2 reaches a speedup of more than 7x when compared to the best multi-core implementation (ClustalW-MPI). In particular, both multi-core approaches present very similar behaviors with differences only appreciable when aligning 300 kb length sequences. On the other hand, the MC64-ClustalWP2 obtains a speedup factor of more than 18x when compared to the original algorithm. The Figures 5 and 6 show the relative acceleration factor in both the first and third stages against ClustalW and ClustalW-MPI, which reveals that our work-distribution strategy for the third stage yields much more parallelism than other methods.

### Variation analyses of the human herpesvirus 1 genomes

As a final test, the MC64-ClustalWP2 was used to run an experiment whose execution time is near prohibitive when non-parallel or less-efficient parallel MSA methods are applied. In this stress test, 37 different human herpesvirus 1 (*Herpes simplex* virus type 1; HHV-1) genomes, publicly available at GenBank <http://www.ncbi.nlm.nih.gov/genbank>, were selected to be aligned. The complete genome of the HHV-1 is about 152 kilobase pairs (kbp), and the different strains should *a priori* present several mutation areas, due to the high mutation-rates of this kind of DNA viruses. Therefore, such alignments can be carried out with dynamic-programming algorithms like Clustal W.

**Figure 7:**
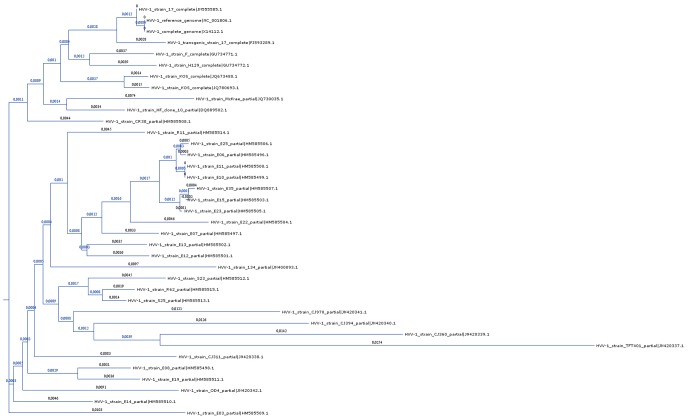
Phylogenetic tree of 37 different genomes of HHV-1. To calculate the multiple alignment of the genomes in this tree, MC64-ClustalWP2 run 7.59 times faster than its closest competitor (ClustalW-MPI).

The MC64-ClustalWP2 aligned the 37 sequence genomes in 36,103 seconds (10.03 h; 21,742 seconds for the first stage, nearly zero for the second one and 14,360 seconds for the third stage). The final phylogenetic tree is shown in Figure 7. The upper part of the dendrogram includes the reference genome, which appears twice in the database (GenBank accession numbers NC_001806.1 and X14112.1) and was modeled from the strain 17 sequencing (GenBank accession number JN555585.1).

The quality of the alignments provided by any MSA algorithm, including Clustal W, depends on many factors, being an issue open for discussion [38], [54]. As expected in this case, the resulting alignment highlighted the polymorphisms between the virus strains. The most divergent strain was the TFT401 ocular one (GenBank accession number JN420337.1), also showing a low sequencing-coverage [55]. The alignment hereby presented confirms the previously reported sequencing results. As an example, a review of these genomes in relation to their glycoproteins coding genes has been published elsewhere [56].

This alignment was also performed with other global MSA algorithms, and even with other Clustal W implementations (which provides exactly the same alignment), but the quickest among them (ClustalW-MPI) required 274,149 seconds (3.17 days) to complete the alignment, being 7.59 times slower than our approach which, hence, becomes the only one capable to deal with this type of alignments in a reasonable time. On the other hand, some of the MSA algorithms do not generate an evolutionary tree of the genomic sequences, as they are oriented to find only the similar regions. Besides, they lack accuracy when the aligned sequences have highly polymorphic regions (e.g., with high mutation-rates), which is typical for most viruses [35].

## Conclusions and Future Work

We have developed a new parallel strategy for Clustal W, which is one of the most relevant algorithms in bioinformatics for multiple-sequence alignments and dendrogram generation. This algorithm uses the pairwise-alignment operation as its core. Our work has focused on large sequences, a field where the resources of a GPGPU architecture have proven insufficient but, in contrast, our previous developments in many-core technologies for bioinformatics, like the MC64-NW/SW, have shown a high performance [20], [22]. In this scenario, we have separately redesigned and parallelized the two most time-consuming stages of Clustal W, using a new approach that exploits parallelism in systems with many cores. This new model, named MC64-ClustalWP2, allows aligning large sequences in a relatively short period of time on a personal computer, obtaining a global speedup of more than 18x against the original Clustal W, and more than 7x against the best x86 parallel implementation to date. This allows aligning more and larger sequences and, thus, enhances the range of problems that can be addressed in an affordable time.

The MC64-ClustalWP2 has been implemented for the heterogeneous standalone system x86-Tile64, using the Tile64 many-core microprocessor. Thus, within a single computer, the MC64-ClustalWP2 offers an impressive performance, being scalable to larger many-core systems using the same parallel strategy. This represents also a proof of concept that new parallel strategies can be exploited to harness the new developments in the many-core microprocessors to analyze the increasing amount of data generated by the second- and third-generation sequencing platforms. Thus, the Clustal W dynamic-programming approach that we have developed (MC64-ClustalWP2) can align genomes with highly polymorphic regions [35], which cannot be accomplished by the MSA heuristic aligners of genomes.

These developments allow to identify identities and differences to generate dendrograms for biodiversity and evolutionary studies, and to develop molecular markers like the ones based, for instance, on Single Nucleotide Polymorphisms (SNP), as well as on microsatellites, also known as Short Tandem Repeats (STR) in animals and as Simple Sequence Repeats (SSR) in plants, for germplasm management, breeding, identification and protection of the intellectual property, PDO, illegal traffic control and fraud prevention, as we have described for the olive oil [57].

Additionally, the MC64-ClustalWP2 strategy can be deployed for any many-core system with very little effort, like the current and future Intel Xeon Phi and the Tilera microprocessors. In particular, we have already developed a threaded version of MC64-NW/SW for the Intel i7 processor, and we are planning to use it as starting point to take full advantage of every Intel Xeon Phi core, where several threads must be executed to achieve an instruction-per-cycle performance. Furthermore, the approach can be escalated, both in processor and in memory resources, interconnecting several TilExpress-20G cards through their 10GBase-CX4 connectors to build a powerful cluster of Tile64 microprocessors, with thousands of cores, increasing both the number and the length of the sequences to align. We have already designed such a cluster, which is currently being evaluated [58].

On the other hand, a possible optimization in order to improve even further the parallelism of the first stage is to get rid of the intermediate Perl script and to transfer its high-level controller functionalities to a core of the many-core system. This allows using a single geometry where each worker is independent (i.e., may indistinctively work for any alignment), receiving in each case the data from the input sequences to be aligned. This new approach would maximize the core usage in the first stage, but would require more data transfers and memory resources in the overall system, which is strongly limited to 8 GB of RAM in the case of the Tile64 hardware. As an additional optimization for the alignment of thousands of sequences, the clustering stage could be parallelized as stated in [59]. However, we are not dealing with such an scenario now, since the MC64-ClustalWP2 is oriented to align long sequences.

On the other hand, the MC64-ClustalWP2 should be considered as a complementary tool to heuristic algorithms like the MGA [33] for the alignment of large sequences with highly polymorphic regions. In this regard, a more complex and “intelligent” alignment system could be built in a heterogeneous environment, in order to distribute and optimize the alignment tasks and stages, attending to the sequence characteristics. These developments should enhance the current tools in the bioinformatics arsenal.

Lastly, the MC64-ClustalWP2 algorithm is freely accessible to the scientific community and can be remotely executed on our server (a system that integrates the TilExpress-20G card) by means of a web service at <http://galactus.uma.es/manycore>. In the same way, the MC64-ClustalWP2 project is available under the GNU General Public License (GPL), and its source code can be downloaded from the same website.
